# A Review of Aspects of Oxidative Hair Dye Chemistry with Special Reference to *N*-Nitrosamine Formation

**DOI:** 10.3390/ma6020517

**Published:** 2013-02-13

**Authors:** David Lewis, John Mama, Jamie Hawkes

**Affiliations:** Perachem Limited, 1 Sizers Court, Henshaw Lane, Yeadon, Leeds LS19 7DP, UK; E-Mails: david@perachem.com (D.L.); john.mama@perachem.com (J.M.)

**Keywords:** hair dye, *N*-nitroso compounds (NOC), nitrosamine, *p*-phenylenediamine (PPD), cancer, risk

## Abstract

This review discusses a new aspect to the safety profile of oxidative hair dyes using data already in the public domain. These dyes contain secondary amines that are capable of forming potentially carcinogenic nitrosamine derivatives when exposed to atmospheric pollution. Numerous scientific articles confirm the existence of secondary amines in hair dyes (and their intermediates), the possibility of nitrosation by atmospheric NO*_x_* of secondary amines to give the *N-*nitrosamines, and the significant safety risks on *N*-nitrosamines. It is believed that such nitrosamine derivatives should be investigated more fully in the interests of consumer safety.

## 1. Introduction

This review heavily references official European Union (EU) opinions and legislation on nitrosamines and hair dye ingredients/products. Worldwide regulations differ only slightly, therefore the arguments put forward are applicable and can be applied to all developed countries.

For a number of years there has been increasing concerns regarding the safety of oxidative hair dyes, or “permanent hair dyes” as they are frequently referred to; many semi-permanent hair dyes are also oxidative. According to Charle and Sag [1] the first oxidative hair dyeing patent can be traced as far back as 1883 when Erdmann and Monnet (French Patent 158,558) used *p*-phenylenediamine (PPD) or toluene-2,5-diamine (PTD) with an oxidizing agent. The use of this dyeing method expanded due to the introduction of new intermediates between 1888 and 1897, which increased the available shade palette; early products included the Ursols and the Furreins produced by the Berlin Aniline Company and the Society of Chemical Industry in Basle respectively [2]. Due to poor performance and serious safety considerations, the technique never gained traction within the textile industry. The technique was, however, used extensively within the fur/hair dyeing industry for many years, due primarily to a lack of alternatives. Whilst the practice of dyeing fur with oxidative dyes has long since been replaced by superior methods, the dyeing of human hair remains almost exclusively oxidative, and although formulations have been modernized the fundamental chemistry remains the same (*i.e.*, the use of aromatic amines as precursors/couplers).

The skin sensitizing nature of hair dye precursors has been well-known for many years. The European Commission Scientific Committee on Consumer Safety (SCCS) (previously known as The European Commission Scientific Committee on Consumer Products, SCCP) has given opinions on 46 hair dye substances, classing 10 as extreme sensitizers (including PPD), 13 as strong sensitizers and 4 as moderate sensitizers [3]. A number of hair dye ingredients are noticeably absent from this list, including toluene-2,5-diamine (PTD) a potent sensitizer, and often used as a replacement for PPD in “PPD-free” oxidative hair dyes. The SCCS Opinion on toluene-2,5-diamine contains data from a number of allergy studies, with positive patch test results as high as 24.8% [4].

Rather than decreasing, allergies to hair dyes have been increasing in incidence in recent years [5], and extreme allergic reactions including coma and even death have been linked to hair dye usage in the media [6,7,8,9,10,11,12].

The first indication that hair dye ingredients may be hazardous to health came in the early 1970s. Shortly after the Ames mutagenicity test was invented, hundreds of commercial products were tested for a biochemistry class experiment conducted by B.N. Ames; only two tested positive: cigarette smoke tar and an oxidative hair dye. In 1975, 169 commercial hair dye formulations were tested [13], and 150 of them tested positive for mutagenicity. This led to a number of hair dye ingredients being banned from use in the 1970s [14].

Whilst the epidemiology evidence is often conflicting, some human studies have reported an increased risk of cancer for permanent hair dye users, including bladder cancer [15,16,17,18,19,20], Hodgkin’s disease [20,21], non-Hodgkin’s lymphoma [21,22,23,24], leukaemia [23,25], breast cancer [26,27,28,29,30], multiple myeloma [21,31], ovarian and brain cancer [32], astrocytoma [33] and brain tumors [34]. Due to the limited data currently available it is not possible to arrive at a definitive conclusion and most studies appear to be limited to a narrow range of cancers, mainly bladder cancer and haematopoietic cancers (lymphoma, Hodgkin’s disease, non-Hodgkin’s lymphoma, leukemia).

A number of studies to date and their conclusions are summarized in SCCS opinions [35,36,37]. Also contained within these SCCS opinions are genotoxicity reports on various hair dye reaction products, submitted by the manufacturers themselves.

## 2. Discussion

This review demonstrates that hair dyes contain secondary amines and that these have the potential to form *N*-nitrosamines when exposed to atmospheric nitrogen oxides. A potential hazard exists, and whilst the risks are currently unknown, the genotoxicity of these compounds should be investigated in the interests of consumer safety in order to ascertain these risks.

It should be noted that a number of textile dyes are also capable of forming *N*-nitrosamines, for example anthraquinone dyes undergo gas fume fading as a direct result of aerial nitrosation [38]. The likelihood of significant nitrosation, however, is low. Very few modern textile dyes have available secondary amines for practical stability reasons, and those that do (the aforementioned anthraquinones) are highly conjugated and stabilized by hydrogen bonds. The same cannot be said for oxidative dyes.

The areas listed below are considered in this review:

2.1. The Chemistry of *N*-nitrosamines
2.2. The Toxicology of *N*-nitrosamines
2.3. Nitrosation of Secondary Amines in Polluted Air
2.4. Secondary Amines in Oxidative Hair Dye Products
2.5. Systemic Activity of Hair Dye Products

### 2.1. The Chemistry of N-Nitrosamines

The term “nitroso compounds” encompasses a broad class of chemicals which have been studied in great detail [39]. In particular, organic *N*-nitroso compounds are of current interest and can be defined as any organic molecule that contains a nitroso group (–N=O) attached to a nitrogen atom. When an amine is exposed to a nitrosating agent, for example nitrous acid, *N*-nitrosamines are produced.

The type of amine is important in determining the final product of the nitrosation [40]:

- Primary alkylamines react, decompose, and yield nitrogen and alcohols. Primary arylamines form the relatively stable diazo compounds which may react further or decompose, depending on the surrounding environment;
- Secondary amines react and form stable *N*-nitrosamines;
- Tertiary amines usually form unstable salts that decompose upon neutralization, or nitrosate away from the nitrogen atom.

**Scheme 1:**
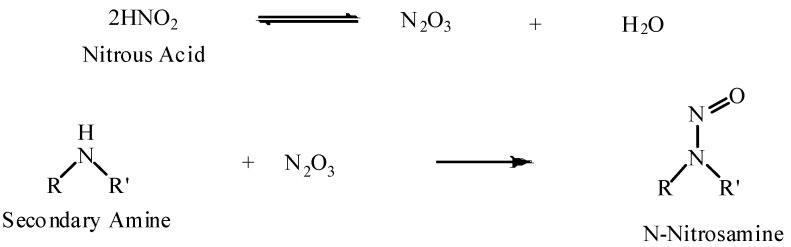
The reaction of a secondary amine with dinitrogen trioxide (via nitrous acid) to form an *N*-Nitrosamine. R, R′ may be alkyl groups, aryl groups or carbon atoms in a ring structure.

Thus, it is the secondary amines that are of importance in the formation of *N*-nitrosamines. Scheme 1 shows the reaction of a secondary amine with nitrous acid to form an *N*-nitrosamine [41].

Nitrosation occurs rapidly in acidic conditions and occurs, albeit at a slower rate, even in strongly alkaline conditions [42]. The speed of the reaction also depends on the nature of the secondary amine to be nitrosated; secondary aromatic amines are more readily nitrosated than simple aliphatic secondary and tertiary amines in acid [43].

Although photodecomposition can occur under acidic conditions, the secondary *N*-nitrosamines are considered chemically stable under physiological and strongly alkaline conditions [44].

### 2.2. The Toxicology of N-Nitrosamines

The toxic and carcinogenic nature of nitrosamines in general has been well established and *N*-nitroso compounds (NOC’s) are considered to be among the most potent carcinogens known. The highly toxic nature of NOC’s was first highlighted by Magee and Barnes in 1956, when it was reported that dimethylnitrosamine produced liver tumors in rats [45]. Since then over 300 nitrosamines and NOC’s have been tested and 90% of them have been found to be carcinogenic in a wide variety of laboratory animals. No species has been found to be resistant against the carcinogenic efficacy of these chemicals and in laboratory studies they have produced cancer in over 39 species (from fish and snake to subhuman primates) [46,47] via all routes of exposure in most vital organs predominately the liver, the oesophagus, the lung, the nasal cavities, the stomach, the kidney, the bladder and the brain [48,49,50].

For the purposes of safety, it is generally considered prudent to assume all NOC’s are carcinogenic unless proven otherwise. The EU Scientific Committee on Consumer Safety (SCCS) in a recent 2012 opinion on Nitrosamines and Secondary Amines in Cosmetic Products specifically states: “When information on a specific NOC structure is not available, the default assumption that all potentially generated NOC will be mutagenic/carcinogenic should be applied.” [51]

The presence of nitrosamines in consumer products, particularly *N*-nitrosamines, is strictly controlled and tight limits are placed on the amounts that may be present or formed. For cosmetics in the EU, the presence of nitrosamines is prohibited under Annex II (410) of the EU cosmetics directive [52].

The general EU purity specifications for cosmetics require an NOC content of not more than 50 parts per billion; additionally, any product that contains amines should not be used with nitrosating systems and be kept in nitrite free environments [51].

### 2.3. Nitrosation of Secondary Amines in Polluted Air

Polluted air contains a number of nitrogen oxides (NO*_x_*), mainly from tobacco smoke, vehicle exhausts, high temperature burners (frequently used to heat buildings), and various other combustion processes. It is these nitrogen oxides that can act as nitrosating agents in the production of *N*-nitrosamines.

This pathway to nitrosamine formation was highlighted in 1972 when Neurath showed that equimolar mixtures of nitrogen dioxide and nitric oxide are capable of nitrosating secondary amines to form highly carcinogenic NOC’s [53]. It was concluded that long term exposure to the levels of nitrogen dioxide present in the atmosphere could pose a significant public health hazard via NOC’s [54].

Whilst *N*-nitrosation in the laboratory is typically carried out under strongly acidic conditions a large amount of research shows nitrosation by NO*_x_* can occur in neutral or even alkaline conditions [55,56,57,58,59,60,61,62,63,64,65]. Moreover, the formation of NOC’s via nitrogen dioxide has been shown to occur *in vivo* in a number of animal studies, either systemically or localized on the skin [66,67,68,69]. It is interesting to note from these aforementioned studies that nitrosation of secondary amines introduced onto the skin still occurs, despite the possibility of competing reactions with the skin itself.

The potential formation of NOC carcinogens on surfaces from atmospheric NO*_x_* exposures was recently highlighted in reference to nicotine residues from tobacco smoke. Residues attached to surfaces were shown to later nitrosate in the presence of atmospheric NO*_x_*. This nitrosated tobacco residue has been dubbed “third hand smoke” and the potential health impact of NOC formation noted [70].

### 2.4. Secondary Amines in Oxidative Hair Dye Products

The following sections deal with the various sources of secondary amines that exist in hair dyes and include:

2.4.1. Oxidative Hair Dye Precursors
2.4.2. Semi-Permanent Hair Color (HC) Dyes (“Direct” Dyes)
2.4.3. Oxidative Reaction Products
2.4.4. Degradation of Oxidative Hair Dyes

#### 2.4.1. Oxidative Hair Dye Precursors

**Chart 1:**
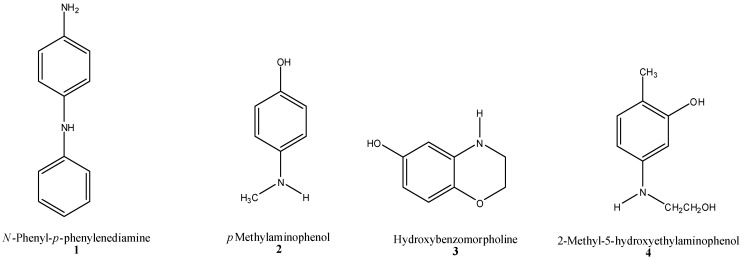

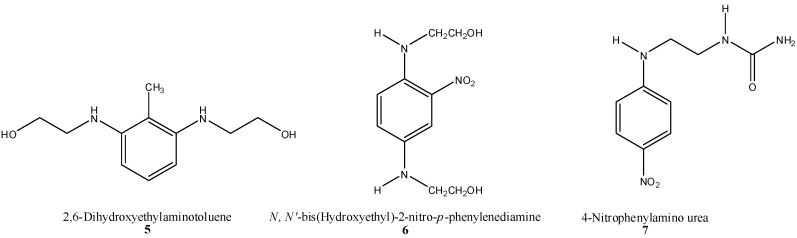
Selected secondary amines present in the EU Cosmetics Directive for use in hair dyes.

Primary, secondary and tertiary amines are ingredients in all oxidative hair dye formulations and are typically used in concentrations of 0.1%–3%. A number of secondary amines were identified in the recent 2012 SCCS nitrosamine opinion [51], seven of which are shown in Chart 1 (1–7). All of these amines can form an *N*-nitrosamine on exposure to nitrosating agents. The compound shown in Structure 7 can form both an *N*-nitrosamine and an *N*-nitrosamide. *N*-nitrosamides are highly unstable in alkaline conditions (e.g., during hair dyeing) and decompose to form powerful alkylating agents, the diazoalkanes, which are highly carcinogenic. Compounds 1–7 are still currently listed in the EU Cosmetics Directive for use in hair dyes.

The SCCS opinion states that these oxidative precursors/couplers can form nitrosamines in the presence of nitrosating agents before and during the hair dyeing process and has therefore restricted the nitrosamine content to <50 ppb. The opinion did not consider that nitrosation of amines can occur after hair dyeing.

These intermediates are only present on the hair/scalp during the dyeing process for a relatively short period of time, thereby limiting human exposure. However, oxidative hair dyeing is an inefficient process and it has been reported that as much as 20%–80% of the applied amines/couplers remain unreacted at the end of a 30 min dyeing application, depending on the combination used [71]. As these intermediates penetrate the hair fiber, it is unlikely that all unreacted intermediates are totally removed during the wash-off procedure. Experience of coloring hair with oxidative dyes shows that the final shade of the dyed hair changes over a 24 h period after dyeing, indicating that intermediates are still present and are further oxidized in air.

These intermediate compounds have a low molecular weight and are fat soluble, thus giving the potential for a significant amount of penetration into the hair and skin to occur. It is unknown how long these intermediates remain on the hair/skin without further testing; however as they are secondary amines they have the potential to form NOC’s.

#### 2.4.2. Semi-Permanent Hair Color (HC) Dyes (“Direct” Dyes)

**Chart 2:**
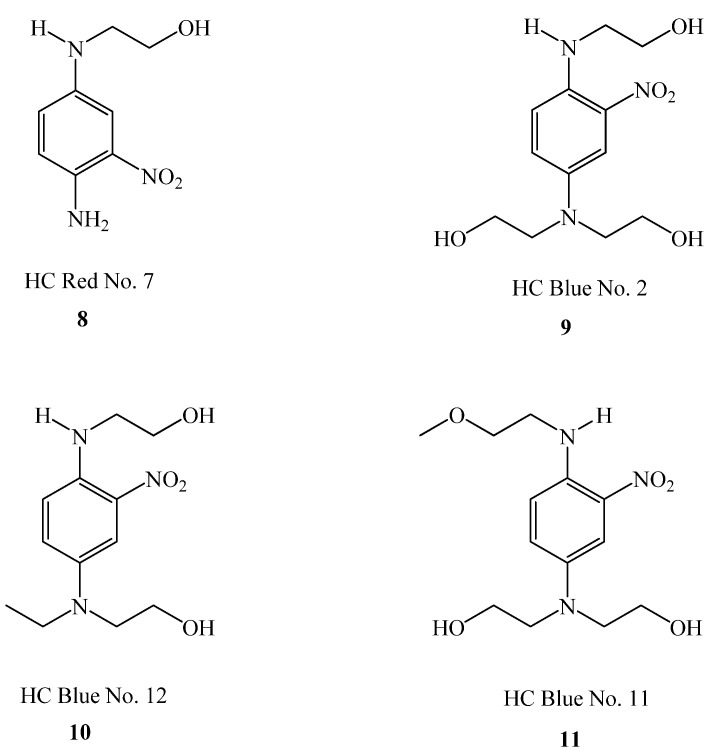
The structures of four commonly used HC dyes.

The HC in HC dyes is sometimes erroneously thought to refer to Hair Color (or Hair Colorants) but does in fact stand for Hemi-Cyanine. These Hemi-Cyanine dyes, sometimes referred to as “Nitro Dyes”, are also a source of secondary amines. The Hair Coloring industry frequently refers to HC dyes as “Direct” Dyes although these should not be confused with the Color Index definition of Direct Dyes. These HC dyes are intrinsically colored and, as well as being used as semi-permanent hair dyes themselves, are often added to “permanent” (oxidative) hair dyes to enhance and broaden the color palette. Four examples of HC dyes are shown in Chart 2 [51]. HC dyes are known to have low extinction coefficients (approximately 10,000 L mol^−1^ cm^−1^) [72], resulting in relatively large amounts being added to hair coloring formulations in order to give a strong visible color. For comparison, anthraquinones are 50% higher at 15,000 L mol^−1^ cm^−1^, mono-azo dyes are typically 30,000 L mol^−1^ cm^−1^ or above, some dis-azo dyes are >60,000 L mol^−1^ cm^−1^ and copper phthalocyanine has an extinction coefficient as high as 100,000 L mol^−1^ cm^−1^.

It can be seen that all the dyes in Chart 2 contain a secondary amine group that can potentially be nitrosated by nitrogen oxides present in the atmosphere. It should also be noted that these compounds are larger molecules than many oxidative precursors (although Structure 1,5–7 are also large), and remain in the hair after the dyeing procedure, hence their “semi-permanent” tag. As the industry itself states, these dyes typically remain in the hair for 6–8 shampoos [73] fading slowly as they leach out and are transferred onto clothes, bedding and the skin.

As was the case for the oxidative intermediates in the previous section, the SCCS opinion [51] revealed that Compounds 8–11 can form nitrosamines in the presence of nitrosating agents, and that the nitrosamine content should be limited to <50 ppb. Again it was not considered that nitrosation of the amines could occur after application.

Four HC dyes were highlighted as secondary amines by the SCCS, however Table 1 shows all the HC dyes currently listed in the EU Cosmetics Directive, those which contain secondary amine groups, and the maximum permitted concentration allowed in the final product (within the EU).

**Table 1:** Hair Color (HC) Dyes currently listed in the current EU Cosmetics Directive, their status as secondary amines and Maximum Permitted Concentration in final product.

Dye	Contains a secondary amine group?	Maximum Permitted Concentration allowed in the final product (%)
HC Blue No. 2	√	2.8
HC Blue No. 7	√	0.68
HC Blue No. 11	√	2.0
HC Blue No. 12	√	1.5
HC Blue No. 13	√	No limit
HC Blue No. 14	√	0.3
HC Blue No. 15	–	NA
HC Blue No. 16	√	No limit
HC Green No. 1	√	Banned
HC Orange No. 1	√	1.0
HC Orange No. 2	√	1.0
HC Orange No. 3	√	Banned
HC Orange No. 5	√	No limit
HC Red No. 1	√	1.0
HC Red No. 3	√	3.0
HC Red No. 7	√	1.0
HC Red No. 8 and its salts	√	Banned
HC Red No. 10	√	1.0
HC Red No. 11	√	1.0
HC Red No. 13	√	2.5
HC Red No. 14	–	NA
HC Red No. 15	√	No limit
HC Red No. 16	√	0.75
HC Violet No. 1	√	0.28
HC Violet No. 2	√	2.0
HC Yellow No. 2	√	0.75
HC Yellow No. 4	√	1.5
HC Yellow No. 7	–	NA
HC Yellow No. 9	√	0.5
HC Yellow No. 10	√	0.1
HC Yellow No. 11	√	Banned
HC Yellow No. 13	√	2.5
HC Yellow No. 14	√	No limit
HC Yellow No. 15	√	No limit

As the table shows, 31 of the 34 HC dyes listed in the EU Cosmetics Directive contain secondary amine groups. The majority of these are of a similar structure to 8–11 (Chart 2), *i.e.*, small molecular weight, fat soluble, aromatic diamines with a nitro group. Six of the dyes are allowed in unlimited quantities, six are allowed to be used at a concentration of 2% or above, and three are currently banned from use.

#### 2.4.3. Oxidative Reaction Products

This section covers both “permanent” and “semi-permanent” hair dyes of an oxidative nature. These two classes of hair dye products share many of the same ingredients and thus have the same propensity to form secondary amines.

**Scheme 2:**
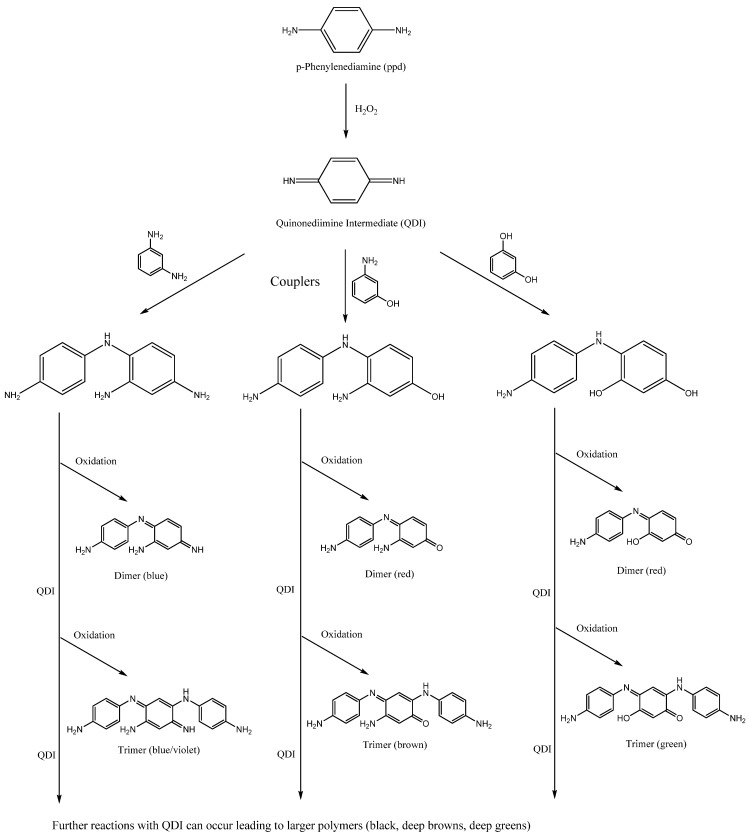
Formation of simple hair dyes from PPD and three different couplers.

The formation of oxidative dyes requires a *para*- or *meta-*substituted aminophenyl precursor (e.g., PPD, PTD, and *m*-phenylenediamine) together with a coupler (e.g., *p*-aminophenol, *m*-aminophenol, and resorcinol). In the presence of peroxide under alkaline conditions, the two chemicals oxidatively couple to give colored molecules. A typical example can be seen in Scheme 2, showing the reaction of PPD with three common couplers and the most likely products [74].

The chemistry involved has been studied extensively [75,76,77,78,79,80,81]. The precursor (PPD in Scheme 2) is oxidized by peroxide to give the QDI intermediate. This intermediate is short lived and reacts rapidly with the coupler(s) to give colorless leuco dyes, which then oxidize to give the hair dye. Both the QDI and the leuco dyes are transitory and do not accumulate during the reaction.

The first step of the reaction is the formation of dimers. As these molecules have a smaller conjugated pathway, the absorption bands are relatively narrow and the colors are typically red/violet/blue. For a deeper color, one with a broader absorption band, trimers or above are required. These species are more brown/green/black. The ratio of dimer to trimer formation depends on the kinetics of the reaction and the structure of the precursors/couplers. If a coupler is used with an ortho substituent, and the precursor is also sterically hindered (e.g., PTD) then it can be appreciated that the reaction will stop at the dimer stage. In most other cases, however, further reaction invariably leads to trimers, and possibly larger structures within the hair. The formation of dimers and trimers is confirmed by industry tests submitted to the SCCS, the results of which can be found in a number of SCCS Opinions [36,37,71,82].

**Scheme 3:**
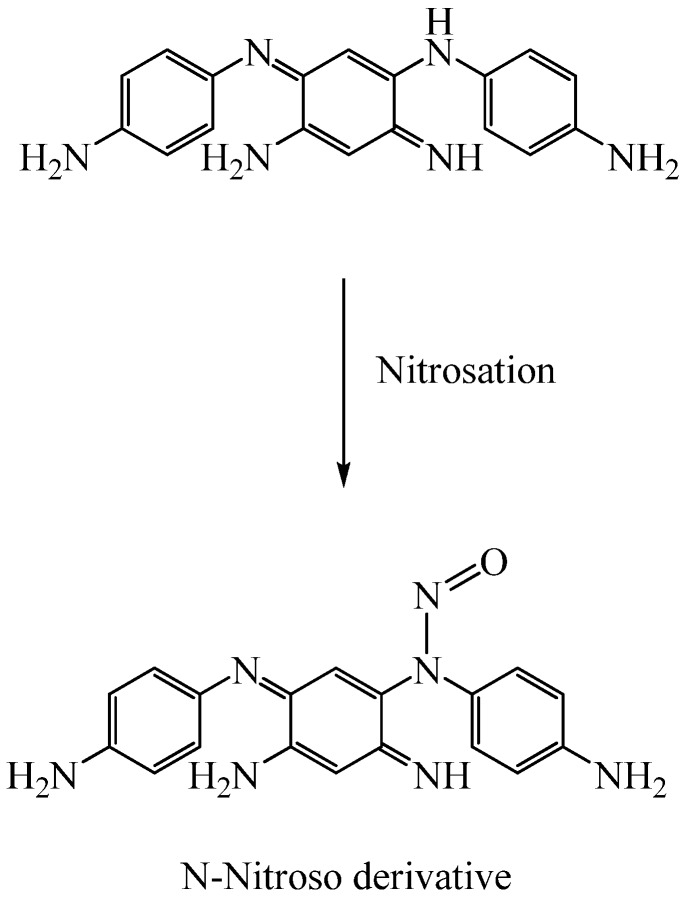
*N*-nitrosation of the secondary amine group in a hair dye trimer.

The structures of the trimer molecules reveal that they all contain an aromatic secondary amino group, which if exposed to a nitrosating agent will form an *N*-nitroso derivative (Scheme 3). Whilst the efficiency of such a transformation is unknown the extent to which it occurs should be investigated.

#### 2.4.4. Degradation of Oxidative Hair Dyes

For most dyed fibers exposed to light, the dye is gradually photooxidized and rendered colorless while at the same time the fiber is photoreduced. The opposite is true for fibers derived from proteins, such as wool, silk and of course hair. In these proteinaceous fibers, it is the dye that is photoreduced and the fiber that is photooxidised [83]. Even in the absence of light, hair contains cysteine thiol and is hence a reducing environment.

It is well known that the azine groups (–N=) in many dye molecules are readily reduced to their secondary amine equivalents, forming a colorless “leuco” compound. Certain dyes are more readily reduced than others, with Oxazines, Thiazines and Azines being particularly susceptible. The products of oxidative hair dyeing can be considered as non-ring-closed azines and are likewise easily reduced. An example is Aniline Black, the oxidative product of aniline, which is readily reduced to the leuco compound [84].

**Scheme 4:**
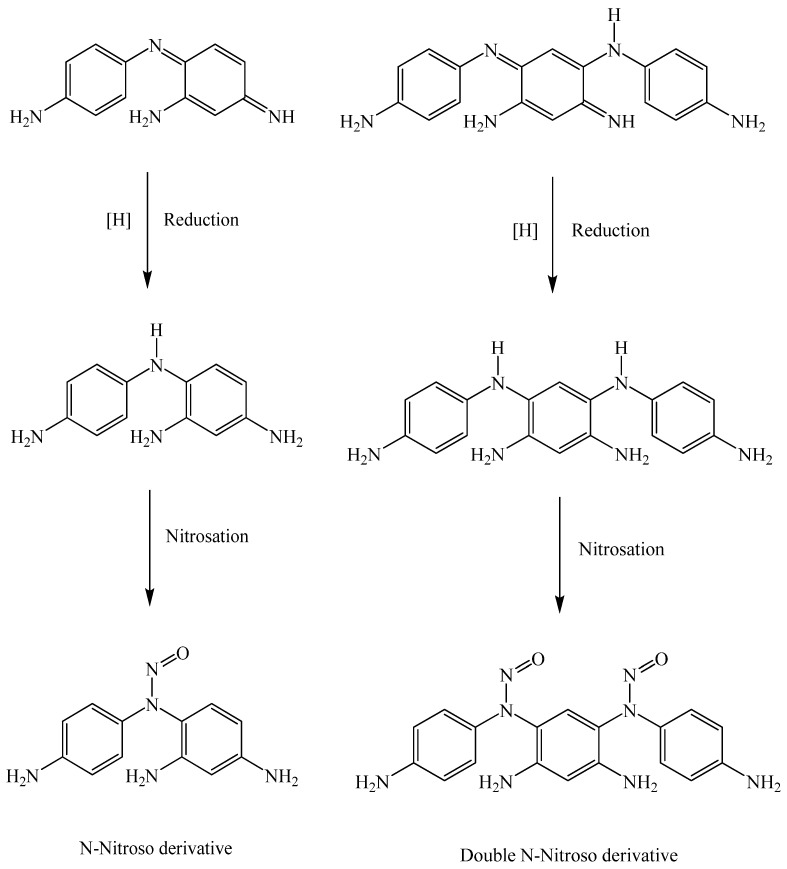
A proposed mechanism for the reduction of a hair dye dimer/trimer and subsequent *N*-nitrosation.

The chemical structures of the dimers/trimers reveal that reduction of the molecule will form the colorless leuco compounds shown in Scheme 4, that these leuco compounds are secondary amines and that they can nitrosate as any other secondary amine would.

During hair dyeing these leuco compounds would be quickly oxidized due to the presence of peroxide, however, after dyeing this peroxide is no longer present. It is likely that the leuco compounds, once formed, would persist in the reducing environment of the hair fiber as secondary amines before air oxidation removes them.

### 2.5. Systemic Activity of Hair Dye Products

Contained within the documentation submitted by the hair dye industry to the SCCS/SCCP [36,37,71,82] is a large amount of data concerning systemic exposure to the hair dye precursors/couplers and reaction products. It was noted in the 2009 Opinion that: “Industry also submitted physico-chemical properties and in vitro dermal absorption studies of nine reaction products of some active ingredients of oxidative hair dyes. The safety evaluation of these performed by SCCP was published in 2006 (SCCP/1004/06) [71]. It was concluded that in some cases significant amounts of oxidative hair dye reaction products become systemically available to the consumer. Studies, similar to those presented, should be extended to include additional indicative combinations of precursors and couplers. According to the updated strategy of hair dyes (genotoxicity, SCCP/0971/06, [85]) further testing may be required.” [36]

Data presented in the above SCCS Opinions shows that starting materials such as PPD can be detected in the urine 48 h after dyeing, highlighting the ability of these species to penetrate into the body. This should raise questions regarding the potential for the corresponding NOC’s also penetrating the body by the same mechanism. The aforementioned SCCS Opinions also present evidence showing that not only are the smaller precursors/couplers capable of penetrating the skin, but the larger dimers and trimers also penetrate, albeit to a lesser degree. It follows then, that if a toxic derivative of these compounds did form, such as an *N*-nitroso compound, then systemic contamination could occur.

Although the oxidative hair dyes are referred to as “permanent”, they do leach out with regular shampooing, and many shades are known to have poor wash-fastness. The oxidative “semi-permanent” dyes are even worse in this respect. Thus, these secondary amines are not “locked-in” the hair shaft as the “permanent” tag may suggest, and therefore any NOC’s formed are also not “locked-in”.

## 3. Conclusions

This review addresses aspects of secondary amine chemistry of oxidative hairs dyes and HC dyes. The potential for secondary amine formation during the oxidative hair dyeing process and during exposure of the dyed hair to light is reviewed.

Nitrosation of the secondary amines is described and conditions to form such NOC’s on the hair are highlighted. In summary the following important points regarding hair dye health and safety are made:

- Secondary amines are produced and are a potential source of NOC’s.
- NOC’s are carcinogenic and even those that have not been tested should be assumed to be just as genotoxic until proven otherwise [51].
- *N*-nitrosation of secondary amines can, and does occur in the atmosphere due to the presence of nitrogen oxides.
- There are a number of sources for secondary amines in oxidative hair products as well as any product using HC dyes, and that these penetrate the skin to become biologically available.

The principles of hair dye chemistry suggest that *N*-nitrosamines may be formed on oxidatively dyed hair. The fact that this possibility exists and does not have appeared to have been investigated merits attention. What is unknown, however, is the amount of NOC’s produced and the potential risk to the consumer these compounds pose; this would need to be determined by further independent research. The ultimate systemic exposure is not currently known, but the SCCS specifically recommend that exposure to nitrosamines should be kept to the “absolute minimum” [86].

Current assessment of risks in hair dyeing are concerned only with the 30 min dyeing time itself, but if secondary amines are present on the hair for months, years, even decades through repeated applications, the risks are clearly magnified. The SCCS notes of guidance on the testing of oxidative hair dye substances for potential genotoxicity [85] do not mention the possibility of NOC formation, or any testing regime for such derivatives. The notes do not consider the possibility of further reactions occurring to the hair dye molecules after the dyeing procedure has completed. It is odd that no recognition of NOC formation from the secondary amines present in hair dye formulations is made, despite the secondary amine limits in other environmental and consumer regulations being specifically designed because of the potential NOC formation.

It is estimated that more than one third of women over age 18 and 10% of men over 40 use some type of hair dye [18]. With the use of oxidative hair dyeing becoming increasingly widespread, it is necessary that the safety issues should be examined in ever more increasing detail.

The SCCS provides an independent advisory role as to whether use of a cosmetic product has been proved safe and recommended for use in a specified type of cosmetic formulation within a defined concentration range. As such, the presence of secondary amines in oxidative (and HC dye based) products should be investigated for *N*-nitrosamine formation on the dyed head in the interests of consumer safety.
